# Supplementary material for: Genomic surveillance of Escherichia coli ST131 identifies local expansion and serial replacement of subclones
Source: Microb Genom. 2020 Mar 26;6(4):e000352. doi: 10.1099/mgen.0.000352 (PMC7276707; doi:10.1099/mgen.0.000352)
Supplement: Supplementary material 1 [file mgen-6-352-s001.pdf]

Supplementary Figures

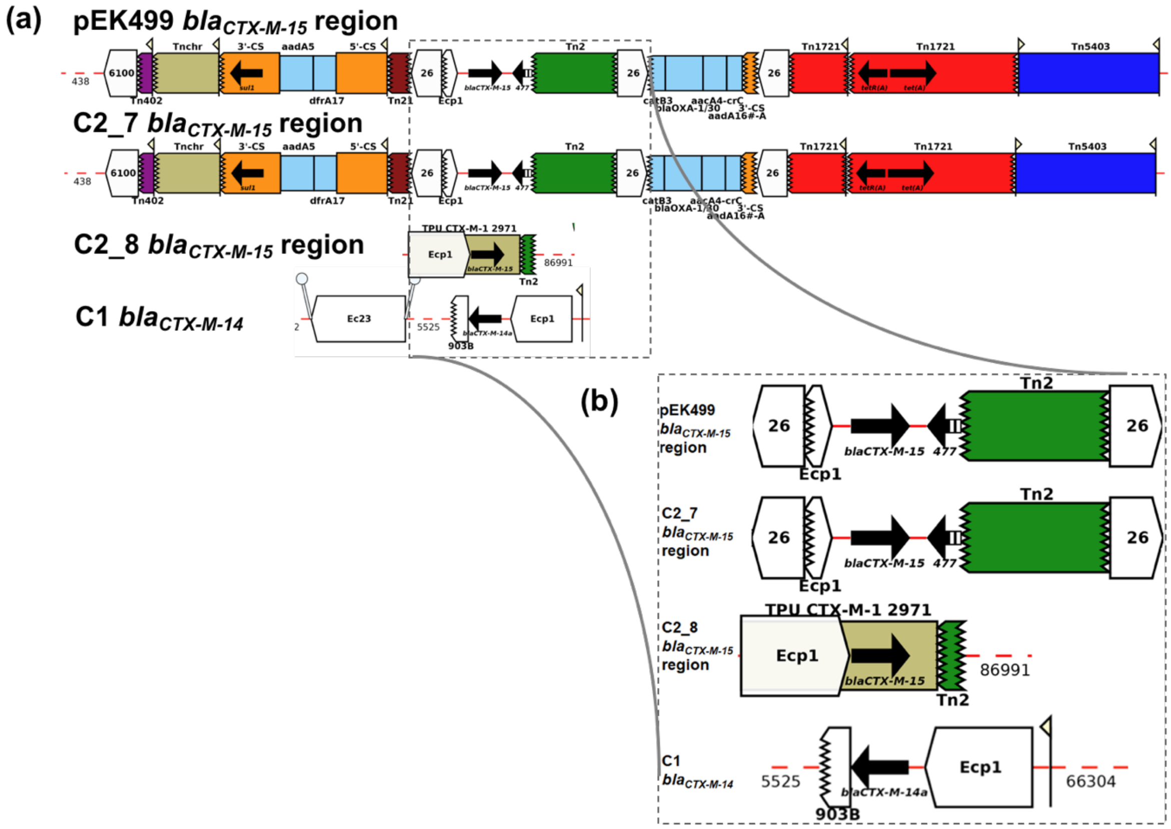

Supplementary Figure 1. Flanking genetic elements of *bla*$_{CTX-M}$ genes in pEK499 and in the chromosome or plasmid-derived contig of C2_7, C2_8 and C1 strains. A *bla*$_{CTX-M-15}$ element on the IncFII/FIA pEK499 reference and in subclade C2_7, a chromosomal *bla*$_{CTX-M-15}$ gene in C2_8 isolates and a *bla*$_{CTX-M-14}$ element on IncFII subclade C1 plasmid (a). The region encoding *bla*$_{CTX-M-14}$ and *bla*$_{CTX-M-15}$ are highlighted in a black box. Arrows indicate the orientation of features, with the forward direction defined as the direction of transcription for genes, towards the main part of the attC site for cassettes, in integrons towards attI for 5' flanking regions, away from the cassette array for 3'-flanking regions, relative to the direction of transcription of the transposase gene for insertion sequences and transposons (Tn) (ie, inverted repeat left to inverted repeat right) and to the direction of the reverse transcriptase for Group II introns. The missing end of a feature is shown by a zig-zag line. The inset represents the area bound by the dashed line and is shown in more detail in (b).

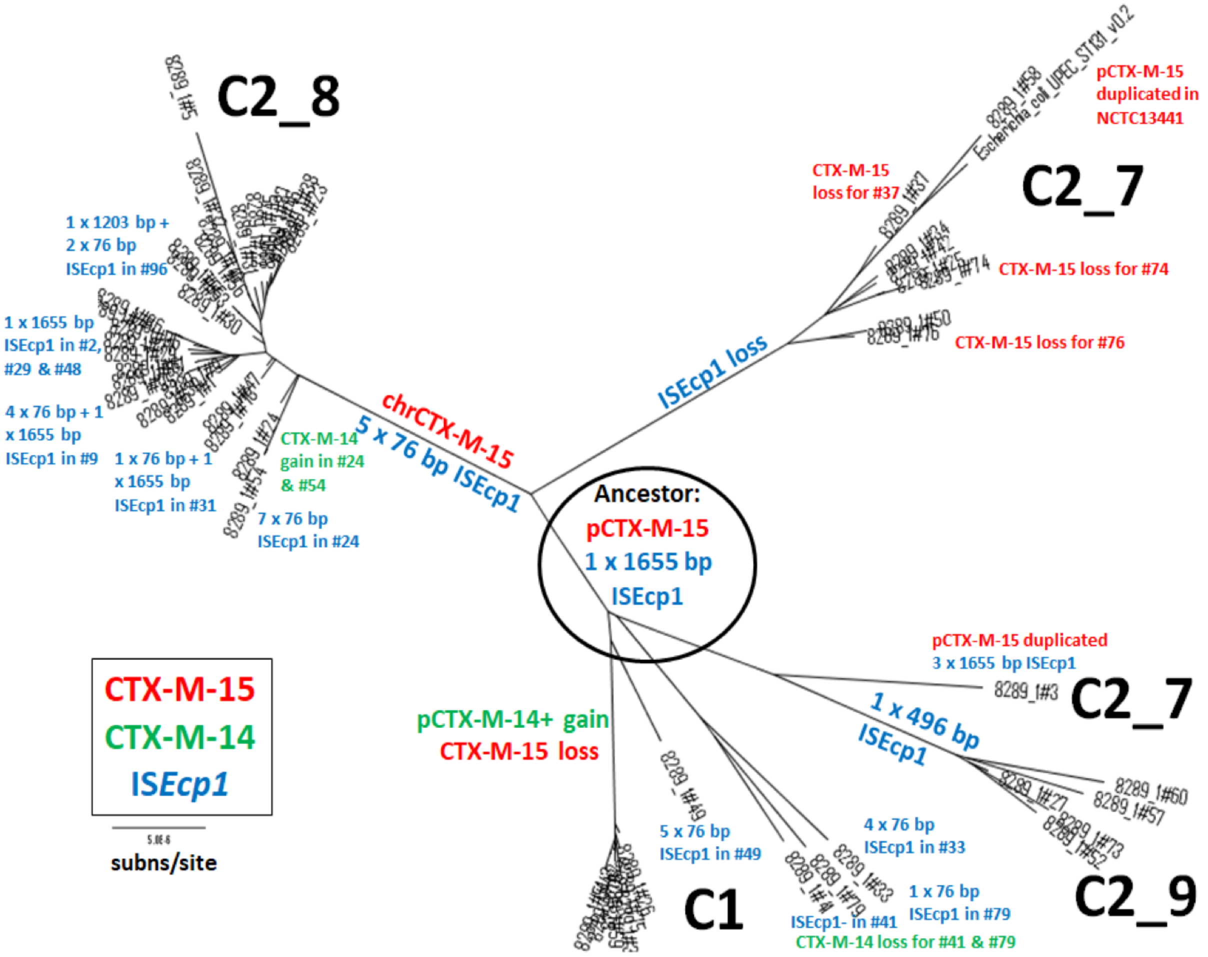

Supplementary figure 2. Phylogenetic reconstruction of N=54 ST131 strains collected from Irish long-term care facilities. A phylogenomic network of the 54 Irish Clade C samples' chromosomal mutational SNPs built using RAxML and ClonalframeML and drawn with FigTree v.1.4.3. The phylogeny of the n=54 was rooted using the topology of n=794, which C1 as the most divergent lineage, with C2_9 diverging next, followed by C2_8 and C2_7, though here the smaller sample size meant that the ancestral lineage was unclear and so could be approximated by the C1-C2 origin, where the ancestor likely had a plasmid with a 1,655 bp IS*Ecp1* 5' of a *bla*$_{CTX\text{-}M\text{-}15}$ gene. The *bla*$_{CTX\text{-}M\text{-}15}$ gene changes are in red, the *bla*$_{CTX\text{-}M\text{-}14}$ gene mutations are in green, and the IS*Ecp1* differences are in blue. The subclades C1, C2_7, C2_8 and C2_9 are shown, and the scale bar shows five substitutions per Mb.

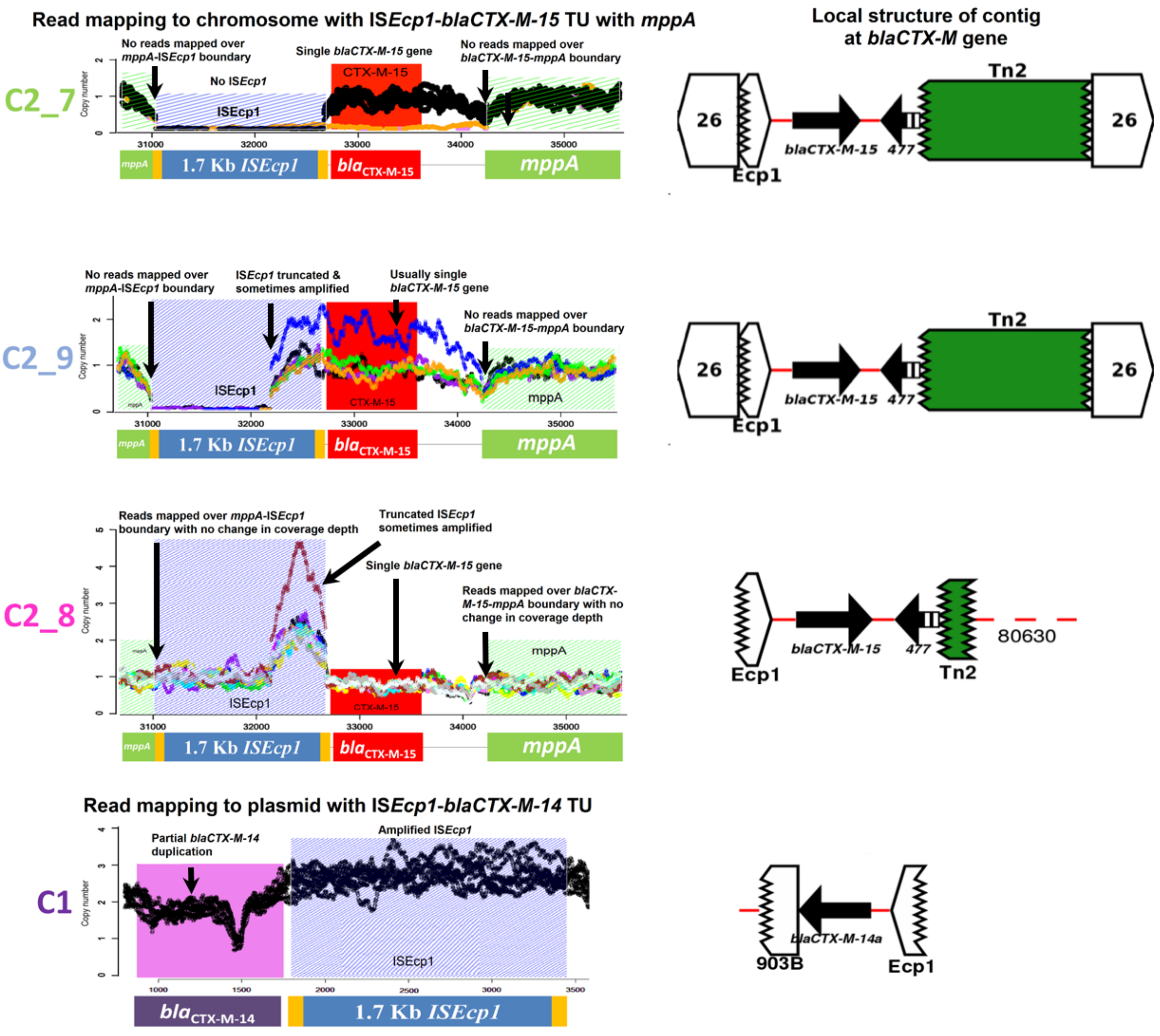

Supplementary Figure 3. Genomic context of *bla*$_{\text{CTX-M}}$ genes in C2_7, C2_9, C2_8 and C1 isolates. Read mapping copy numbers for 33 of the 54 the Irish isolates from C2_7 (n=6 shown), C2_9 (all n=5 shown), C2_8 (n=15 shown) and C1 (n=7 shown) across IS*Ecp1* elements (blue), the *bla*$_{CTX\text{-}M\text{-}15}$ gene (red), the *bla*$_{CTX\text{-}M\text{-}14}$ gene (pink) or the *mppA* gene (green). C2_8 all had consistent coverage of the chromosomally inserted TU isoform IS*Ecp1-bla*$_{\text{CTX-M-15}}$-shortTn*2* spanning *mppA* and typically had with IS*Ecp1* fragments of 1,203 bp, 529 bp, 76 bp and 76 bp. The C2_9 isolates had a 496 bp IS*Ecp1* element and a *bla*$_{CTX\text{-}M\text{-}15}$ gene. Most C2_7 isolates had no IS*Ecp1* and one *bla*$_{CTX\text{-}M\text{-}15}$ gene. C1 had the TU isoform p_IS*Ecp1-bla*$_{\text{CTX-M-14}}$-IS*903B* with a duplicated IS*Ecp1* element and duplicated *bla*$_{CTX\text{-}M\text{-}14}$ gene. Reads from non-C2_8 libraries mapped at *mppA* in the TU isoform, but with gaps indicating no contiguous mapping to the *bla*$_{CTX\text{-}M\text{-}15}$ gene.

(a)

(b)

(c)

(d)

(e)

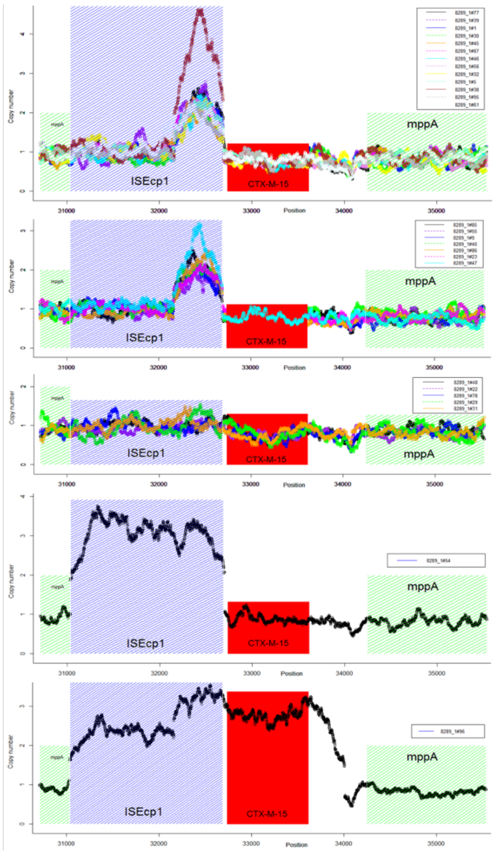

Strain	ISEcp1-1203bp			ISEcp1-529bp			ISEcp1-76bp			CTM15	
#77	2515747	2516949	101	2517050	2517578	102	2517680	2517755	50	2517805	2518681
#39	2463781	2464983	101	2465084	2465612	102	2465714	2465789	50	2465839	2466715
#1	2517655	2518857	101	2518958	2519486	102	2519588	2519663	50	2519713	2520589
#30	2522218	2523420	101	2523521	2524049	102	2524151	2524226	50	2524276	2525152
#45	2507955	2509157	101	2509258	2509786	102	2509888	2509963	50	2510013	2510889
#87	2513858	2515060	101	2515161	2515689	102	2515791	2515866	50	2515916	2516792
#46	2486010	2487212	101	2487313	2487841	102	2487943	2488018	50	2488068	2488944
#56	2493709	2494911	101	2495012	2495540	102	2495642	2495717	50	2495767	2496643
#32	2492145	2493347	101	2493448	2493976	102	2494078	2494153	50	2494203	2495079
#5	2483954	2485156	101	2485257	2485785	102	2485887	2485962	57725	2543687	2544563
#38	2514489	2515691	101	2515792	2516320	27242	2543562	2543637	50	2543687	2544563
#95	2513089	2514291	101	2514392	2514920	32815	2547735	2547810	50	2547860	2548736
#61	2515094	2516296	101	2516397	2516925	6835	2523760	2523835	50	2523885	2524761

Strain	ISEcp1-1203bp			ISEcp1-76bp			CTM15	
#85	2514846	2516048	101	2516149	2516224	50	2516274	2517150
#55	2512460	2513662	101	2513763	2513838	50	2513888	2514764
#9	2516536	2517738	101	2517839	2517914	50	2517964	2518840
#40	2515338	2516540	101	2516641	2516716	50	2516766	2517642
#86	2514655	2515857	101	2515958	2516033	50	2516083	2516959
#23	2513116	2514318	101	2514419	2514494	50	2514544	2515420
#47	2507474	2508676	101	2508777	2508852	50	2508902	2509778

Strain	ISEcp1-1655bp			CTM15	
#48	2504370	2506024	50	2506074	2506950
#22	2495270	2496924	50	2496974	2497850
#78	2524663	2526317	50	2526367	2527243
#29	2499422	2501076	50	2501126	2502002
#31	2504151	2505805	50	2505855	2506731

Strain	ISEcp1-76bp			ISEcp1-74bp			CTM15	
#54	2514328	2514403	101	2514504	2514577	50	2514627	2515503

Strain	ISEcp1-1203bp			ISEcp1-529bp			CTM15	
#96	2513914	2515116	2584923	5100039	5100567	50	5100617	5101493

Supplementary Figure 4. C2_8 isolates with a chromosomal IS*Ecp1-bla*$_{CTX\text{-}M\text{-}15}$ at the *mppA* gene. Read mapping copy number for n=27 C2_8 isolates showing that all had 1+ IS*Ecp1* elements (blue) 5' of a *bla*$_{CTX\text{-}M\text{-}15}$ gene (red) with consistent coverage spanning the *mppA* gene (green) including reads spanning each elements' breakpoints, with the TU isoform chr_shortIS*Ecp1-bla*$_{CTX\text{-}M\text{-}15}$-shortTn*2*. N=13 (a) had IS*Ecp1* fragments of 1,203 bp, 529 bp, 76 bp and 76 bp with one *bla*$_{CTX\text{-}M\text{-}15}$ gene, though within this some had one or two extra 76 bp IS*Ecp1* fragments on their plasmids, including 8289_1#24 (ERR191657 in Table 3, not shown here) and 8289_1#53 (not shown here). 8289_1#24 had a *bla*$_{CTX\text{-}M\text{-}14}$ gene, and had 74-77 bp IS*Ecp1* fragments on its plasmid, as well as 76 bp, 529 bp and 1203 bp IS*Ecp1* segments on its chromosome. 8289_1#5 (ERR191638) also had its *bla*$_{CTX\text{-}M\text{-}15}$ gene was 57,725 bp distant from the IS*Ecp1* fragments. 8289_1#38 (ERR191671), 8289_1#61 (ERR191694) and 8289_1#95 (ERR191728) had a 76 bp IS*Ecp1* fragment adjacent to the chromosomal *bla*$_{CTX\text{-}M\text{-}15}$ gene at 27,742 bp (8289_1#38), 6,835 bp (8289_1#61) and 32,815 (8289_1#95) from the other IS*Ecp1* copies, consistent with recombination between IS*Ecp1* segments. N=7 (b) were like this previous group, but without the 529 bp IS*Ecp1* fragment. Another set (n=5, third panel) had a full 1,655 bp IS*Ecp1* element with no fragmentation. One isolate (8289_1#54) had three full 1,655 bp IS*Ecp1* elements on both its chromosome and plasmid, and fragments of 76 bp and 74 bp adjacent to the *bla*$_{CTX\text{-}M\text{-}15}$ gene (c). Finally, the strain ERR191729 (8289_1#96) had three *bla*$_{CTX\text{-}M\text{-}15}$ gene copies and a first IS*Ecp1* fragment 529 bp where the TU was inverted and duplicated, and separate from a 1,203 bp IS*Ecp1* at mppA, suggesting that recombination between the chromosomal and plasmid IS*Ecp1* IRs may have transferred the *bla*$_{CTX\text{-}M\text{-}15}$ gene back to the plasmid (e). The tables (right) shows the IS*Ecp1* assembly coordinates, spacer DNA lengths and *bla*$_{CTX\text{-}M\text{-}14}$ assembly coordinates.

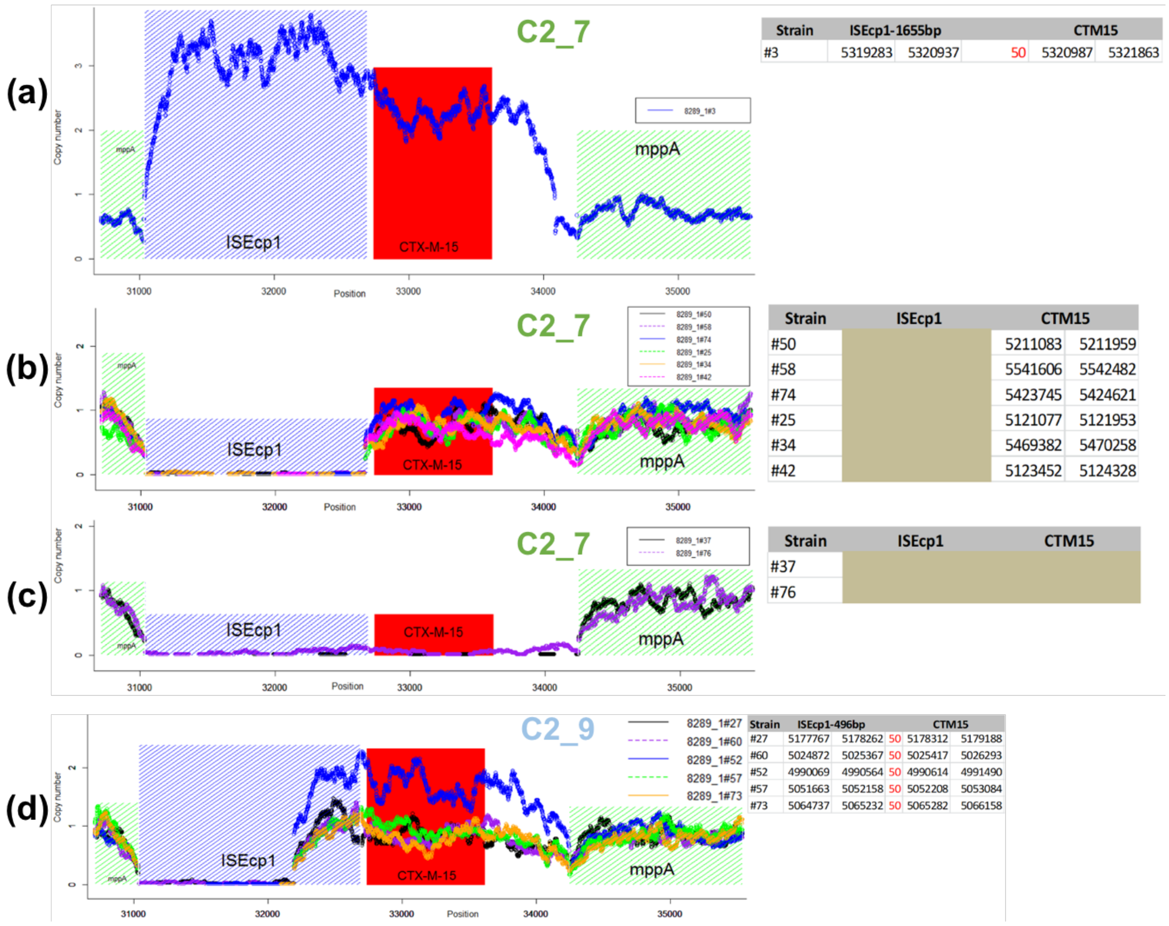

Strain	ISEcp1-1655bp			CTM15	
#3	5319283	5320937	50	5320987	5321863

Strain	ISEcp1	CTM15	
#50		5211083	5211959
#58		5541606	5542482
#74		5423745	5424621
#25		5121077	5121953
#34		5469382	5470258
#42		5123452	5124328

Strain	ISEcp1	CTM15
#37		
#76		

Strain	ISEcp1-496bp			CTM15	
#27	5177767	5178262	50	5178312	5179188
#60	5024872	5025367	50	5025417	5026293
#52	4990069	4990564	50	4990614	4991490
#57	5051663	5052158	50	5052208	5053084
#73	5064737	5065232	50	5065282	5066158

Supplementary Figure 5. C2_7 and C2_9 strains with a plasmid-bound IS*Ecp1*-*bla*$_{CTX\text{-}M\text{-}15}$. Read mapping copy number for n=9 C2_7 and n=5 C2_9 isolates showing one from C2_7 (8289_1#3, ERR191636, (a)) had a three IS*Ecp1* copies (blue) and a duplicated *bla*$_{CTX\text{-}M\text{-}15}$ gene (red), but no contiguity if reads mapping across to *mppA* (green). These had the TU isoform p_*bla*$_{CTX\text{-}M\text{-}15}$-orf477Δ-Tn*2*. The majority of C2_7 (n=6, (b)) had no IS*Ecp1* and one *bla*$_{CTX\text{-}M\text{-}15}$ gene. Two C2_7 isolates (c) had no IS*Ecp1* and no *bla*$_{CTX\text{-}M\text{-}14}$ gene. All n=5 C2_9 isolates (d) had a 496 bp IS*Ecp1* element (blue) 5' of a *bla*$_{CTX\text{-}M\text{-}15}$ gene (red), but no contiguity if reads mapping across to *mppA* (green) unlike C2_8. One (8289_1#52, blue) had a partial amplification of this TU. The tables on the right show the IS*Ecp1* assembly coordinates, 50 bp spacer length and *bla*$_{CTX\text{-}M\text{-}14}$ assembly coordinates.
